# Sexual attraction modulates interpersonal distance and approach-avoidance movements towards virtual agents in males

**DOI:** 10.1371/journal.pone.0231539

**Published:** 2020-04-21

**Authors:** Robin Welsch, Christoph von Castell, Martin Rettenberger, Daniel Turner, Heiko Hecht, Peter Fromberger

**Affiliations:** 1 Department of Psychology, Johannes Gutenberg-University Mainz, Mainz, Germany; 2 Centre of Criminology Wiesbaden, Wiesbaden, Germany; 3 University Medical Center of the Johannes Gutenberg University Mainz, Mainz, Germany; 4 Human Medical Center, Clinic for Psychiatry and Psychotherapy, Forensic Psychiatry, Göttingen, Germany; Rice University, UNITED STATES

## Abstract

How does sexual attraction alter social interaction behavior? We examined the influence of sexual orientation on locomotor approach-avoidance behavior and interpersonal distance. We immersed androphilic and gynophilic male subjects into a virtual environment and presented various male and female virtual persons. In the first experiment, subjects took a step forward (approach) or backward (avoidance) in response to the sex of the virtual person. We measured reaction time, peak velocity, and step size, and obtained ratings of sexual attractiveness in every trial. In the second experiment, subjects had to approach the virtual person as if they were to engage in a social interaction. Here, we analyzed interpersonal distance and peak velocity of the approaches. Our results suggest that sexual attraction facilitates the approach response and reduces the preferred interpersonal distance. We discuss our findings in terms of proxemics, current findings in sex research, and the applicability of our novel task in other fields of psychological research.

## Introduction

We feel attraction towards stimuli that we desire and a repulsion from stimuli that we detest. Positively evaluated stimuli elicit an approach reaction, whereas negatively evaluated stimuli trigger avoidance behavior [1]. For example, interpersonal attraction can be described along the dimension of approach and avoidance, which in turn relates to smaller or larger interpersonal distances (IPD) in proximity tasks [2, 3].

One of the most important current theories on human sexuality proposes that sexual arousal depends on the individual responsiveness of two distinct neurophysiological systems: sexual excitation and sexual inhibition [4]. The so-called Dual Control Model (DCM) makes three further assumptions: (1) individuals vary in their propensity for excitation and inhibition, (2) these excitatory and inhibitory responses are mostly adaptive and functional, and (3) excitation causes approach behavior towards sexually arousing stimuli, whereas inhibition supports avoidance behavior towards sexually non-arousing stimuli [5–7]. Interestingly, the influence of sexual stimuli on locomotor responses in social interactions (i.e., body movements towards the sexually arousing stimuli) has rarely been subject to experimental research in humans. Locomotor approach behavior should be affected by sexually arousing stimuli: the higher the sexual arousal related to a stimulus, the more distinct the locomotor approach behavior, i.e., one should approach sexually arousing stimuli closer, faster, and more immediately than sexually non-arousing stimuli.

### Approach-avoidance

The approach and avoidance reaction has first been measured by Solarz [8]. He presented words of either positive (e.g., “happy”) or negative valence (“stupid”). Subjects then reacted with a previously learned set of arm movements as fast as possible. In compatible trials, subjects reacted with the push of a lever away from the body to words of negative valence, which constituted an avoidance reaction, and with a pull of the lever to positive words (approach reaction). In incompatible trials, the mapping of valence and reaction direction was reversed (push—positive, pull—negative). The results of this experiment showed that compatible trials produced faster reaction times (RTs) as compared to incompatible trials.

Variants of this Approach-Avoidance Task (AAT) have been implemented in different contexts including sex research [7, 9–11]. For example, Hofmann, Friese [12] presented sexual and artistic stimuli to gynophilic men. In a first block, subjects were instructed to push a joystick when seeing a sexual stimulus and to pull the joystick when an art stimulus was depicted. The assignment was reversed in the next block. Their findings suggested a larger compatibility effect for sexually arousing stimuli compared to art stimuli. This approach-effect towards sexually relevant stimuli has been partly replicated in (child) sexual offenders [10] and has been linked to sexual excitation and arousal among gynophilic men [7, 13]. However, the supposed interaction of movement direction and sexual orientation towards the target stimulus did not surface consistently [7, 10].

### Interpersonal distance

Physical proximity is regulated as a function of approach and avoidance forces, to an appropriate level of interpersonal intimacy or psychological distance [14]. Hence, it is obvious that romantic partners keep closer IPDs than do friends or strangers [15]. In non-acquainted pairs, two males keep a greater distance from each other than mixed sex pairs, and female pairs prefer shortest distances [for a review see 16]. However, the mechanisms underlying sex effects on IPD are not well understood, potentially due to the problem of independently varying factors such as gender or sexuality in a controlled experimental setting. The latter may also account for the heterogeneous findings concerning the size of the IPD sex effect.

Interestingly, Uzzell and Horne [17] have identified that not biological sex per se but rather sexual identity and sexual orientation determine the sex effect on IPD. For purposes of better experimental control, some proxemic researchers have chosen to use virtual environments with virtual persons [18]. In virtual environments, sex effects on IPD are sometimes present [19], sometimes absent [20], and sometimes not modeled at all [2]. With careful choice and good rendering quality of the avatars such effects should surface, if present at all.

In sum, we suggest that IPD as measured in a virtual environment may reflect the attraction between interactants as an end to approach and avoidance-related behavior. The previously reported sex effect on IPD, male-male > mixed > female-female, may be influenced by sexual orientation and sexual relevance of the target.

### Aims of the study

The primary goal of this study was to investigate how sexual attraction modulates approach/avoidance-related behavior in social interaction. We did this by administering an AAT as well as an IPD-paradigm. Previous studies in the domain of sexually motivated behavior have either considered AAT response times to pictorial stimuli or IPD as measures of approach/avoidance-related behavior, but they never considered both paradigms simultaneously. We hold that this is necessary as both paradigms have different serious shortcomings, which make conclusions based on just one paradigm problematic. The former approach (AAT) suffers from a lack of ecological validity, the latter approach (IPD) is often plagued by confounding variables and a limited number of sexually relevant stimuli [10, 17]. We sought to address these drawbacks by using a dual-paradigm virtual-reality approach. This allowed us to present a range of sexual stimuli in an ecologically valid social interaction scenario. Considering that the AAT and the IPD-paradigm have not yet been implemented in virtual reality to study sexually motivated behavior, our second goal was to conceptually replicate and extend previous findings, e.g. by considering effects on other dependent variables such as the approach speed when engaging in a social interaction.

Third, we consider for the first time two different ways of examining sexual attraction to a stimulus. Either one can indirectly infer sexual attraction from the sexual orientation of the subject (androphilic subjects are supposedly more attracted to all male stimuli, gynophilic subjects are more attracted to all female stimuli) or one can directly request subjects to rate the sexual attractiveness of stimuli. Previous studies have used the former approach, which may have contributed to mixed findings in the literature. The latter approach allows for individual variation in the attraction towards a male or a female stimulus and collapses two experimental factors into one, which potentially increases statistical power. By combining these two approaches, we tried to make use of the benefits of both approaches.

From a more theoretical point of view, spatial and temporal components of approach and avoidance behavior, such as RT and IPD, may be intertwined. Equilibrium theory [14, 21] suggests that approach-avoidance motivation regulates IPD. Therefore, RT differences in the AAT should relate to IPD. Previous studies could show that a facilitated avoidance response indeed relates to a preference for larger IPD [22], the reverse effect however, a propensity to approach promoting smaller IPD, has not yet been tested. Therefore, our fourth goal was to directly compare approach/avoidance-related behavior in the AAT and the IPD-paradigm within subjects.

For the AAT Experiment, we hypothesized that the approach reaction as compared to the avoidance reaction is facilitated when sexually attractive stimuli are presented. Recent studies suggest that visual flow increases approach-avoidance effects in a joystick task [23], and that whole-body forward/backward movements can serve as approach-avoidance motor reactions [24]. Hence, we chose to implement an AAT in which subjects were instructed to step forward or backward while engaging in whole-body approach-avoidance movements towards more or less attractive virtual persons. Comparable to Stins, Roelofs [24] we parametrized approach and avoidance locomotor reactions in terms of distance and time by extracting the reaction initiation time, step size, and peak velocity from the tracking data.

In the IPD Experiment, subjects engaged in social encounters with male and female virtual persons while we tracked their movements within the virtual environment. We hypothesized that sexually attractive stimuli produce shorter IPDs than sexually unattractive stimuli. We expected that subjects, irrespective of sexual orientation, prefer larger distances towards male virtual agents as compared to female virtual agents [20]. Following Uzzell and Horne [17], we hypothesized that this IPD sex effect is reduced in androphilic subjects, due to relatively stronger attraction between subject and avatar (as measured by peak velocity extracted from the tracking data). Furthermore, we expected that IPD relates to the facilitation of approach movements in response to sexually attractive avatars.

AAT research has not shown any order-effects with regard to approach-avoidance biases [see 23, 25] whereas IPD can be affected by familiarity of the approached person [16, 26]. To minimize potential effects of stimulus-exposure and habituation, all subjects first completed the IPD Experiment and then the AAT Experiment. That is, we presented the IPD Experiment before the AAT Experiment rather than splitting the groups and counterbalancing the order, to minimize potential within-experiment variability.

## AAT experiment

To examine the influence of sexual attraction on the approach and avoidance response we developed a AAT where male subjects had to react with a step forward or a step backward in response to the sex of the avatar. In one block, subjects were requested to perform an approach reaction, stepping forward, in response to a female stimulus and an avoidance reaction, stepping backward, in response to a male stimulus and in the other block to follow a reversed instruction (avoid—female; approach—male).

### Method

#### Subjects

79 male volunteers took part in the study, 7 subjects were excluded from the statistical analyses: One subject was identified as being bisexual and six subjects produced IPDs well above 1.6 m in the IPD Experiment, which indicates that they did not comply with the task instruction to engage in a conversation. The remaining 72 subjects (24 of whom were androphilic) were 19 to 36 years old (*M* = 24.97, *SD* = 4.21). All remaining subjects completed both Experiments. Subjects were recruited via advertisements on the campus of the University of Mainz and the University of Göttingen and in associated online communities. All subjects had normal or corrected-to-normal visual acuity (Snellen fraction 6/6 or better) as determined by the Freiburg Visual Acuity Test [FrACT; 27]. Stereoscopic acuity was tested using a digital version of the Titmus Test [28], which presented 9 stimuli with stereoscopic disparities of 800, 400, 200, 140, 100, 80, 60, 50, and 40 seconds of arc respectively. The criterion for participation was that at least six of the nine trials of the Titmus Test had been answered correctly. All subjects reported to be right-handed. Subjects did not receive any monetary compensation but could obtain partial course credit for participation in the study.

To control for potential effects of nationality and culture [29, 30] we verified that all subjects had a German cultural background as indicated by their German citizenship. We chose to sample andro- and gynophilic male subjects for two reasons: First, reactions towards visual sexual stimuli are more attenuated in male as compared to female subjects [31], thus males should produce larger effects. Second, sex research using the AAT has mostly relied on male subjects [7, 10, 12].

Sexual orientation was assessed using the Kinsey scale [30], on which subjects reported their sexual orientation reaching from 0 (solemnly heterosexual) to 6 (solemnly homosexual). As common practice in sex research [7, 32–34], we formed two groups by considering exclusive and predominantly exclusive homosexual orientation and heterosexual orientation. 48 subjects chose 0 or 1 on the Kinsey Scale, which can be categorized as a heterosexual orientation and thus gynophilic, and 24 subjects chose 5 or 6 on the Kinsey scale, which indicates a homosexual orientation and thus androphilia.

#### Apparatus and stimuli

Subjects saw stereoscopic full-scale simulations on an HTC Vive head-mounted display (1080 x 1200 pixels per eye and a refresh rate of 90 Hz). The field of view (FOV) was approximately 100 degrees vertically by 110 degrees horizontally. Both the head-mounted display and the controller were tracked with a sampling frequency of about 85 Hz. Subjects’ individual inter-pupillary distance was measured by means of a pupil-distance meter and taken into account when computing the stereoscopic disparity of the virtual environment, which resembled the surrounding laboratory. The subjects’ movement was tracked with 120 Hz.

Stimuli were presented using the virtual environment software Vizard 5 [35]. Avatars were chosen from the Complete Characters HD Set (Rocketbox Studios GmbH, Hannover, Germany). To control for potential effects of gaze direction [14, 21, 36], the avatar’s eyes were dynamically adjusted so that they looked directly onto the observer’s bridge of the nose. The subject was positioned in front of the avatar, facing it directly. Initial distance was set at 150 cm from the avatar.

#### Design and procedure

**Fig 1:**
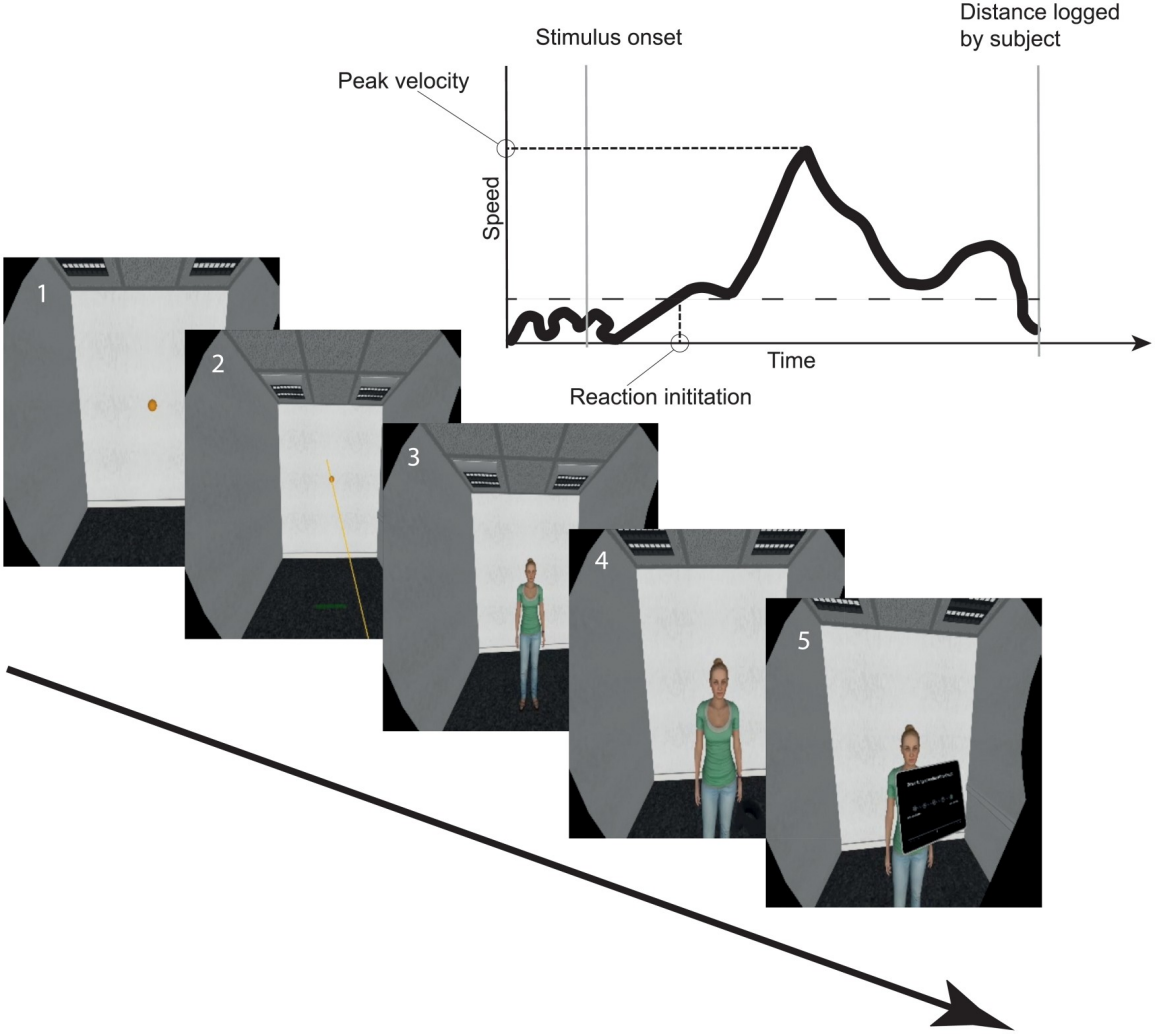
Sample trial from the AAT Experiment. Bottom: Screenshots of one example trial: (1) presentation of the sphere, (2) the subject aiming at the sphere, (3) stimulus onset, (4) the subject approaching the avatar, and (5) rating of the avatar. Top: schematic representation of a velocity curve within one trial.

In the AAT Experiment, we varied two experimental factors within subjects: avatar sex (10 male, 10 female) and reaction direction (i.e., mapping of approach and avoidance). Each of the stimuli was to be approached and avoided four times in response to the sex of the avatar, resulting in 160 trials. At the beginning of each trial, subjects were presented with a sphere at approximately their own height (see Fig 1, panel 1). After subjects had aimed at the sphere with the controller (see Fig 1, panel 2), the avatar appeared (see Fig 1, panel 3), and the subjects stepped forward or backward (see Fig 1, panel 4). In one block, subjects avoided the female avatar by stepping backward and approached the male avatar by stepping forward. In the other block, this mapping was reversed, and subjects saw the same set of stimuli again. Subjects were instructed to react as quickly and correctly as possible. Afterwards, the subject judged the avatar’s sexual attractiveness on a 5-point rating scale (1 = *sexually unattractive to* 5 = *sexually attractive*). The scale was depicted on a virtual handheld device on which subjects could interactively choose a value on the scale using the HTC VIVE controller (see Fig 1, panel 5). Then the avatar disappeared, and the subject went back to the starting position at 150 cm distance from the avatar. No time limit was given. Subjects were instructed both in written and verbal form. The order of blocks was counterbalanced between subjects and trials were presented in random orders. Subjects completed 10 training trials prior to every block.

In accordance with the Declaration of Helsinki, subjects gave written informed consent and were debriefed after the experiment. Questionnaires were filled out in a paper-pencil format after the experimental tasks. Subjects gave general demographic information such as age, gender, and education as well as information concerning personal well-being and current sexual functioning. Participation in the whole study took about 90 minutes. The study was approved by the ethics committee of the University of Göttingen.

#### Data preparation and statistical analysis

We obtained translational kinematic data from the HTC VIVE Headset. It had an effective sampling frequency of about 85 position logs per second and produced on average 207 XYZ data points per subject per trial at an average trial duration of 2440 ms. In each trial, head position was recorded from stimulus onset until the subject started the stimulus rating. To smoothen the tracking data, we applied a Salvitzky-Golay filter (window = 11, polynom = 2) using the trajr package [37] in R [38]. Next, we calculated movement speed for every time frame and set a cut-off of 10 cm/s to separate body movement from body sway. Then we extracted relevant movement parameters such as reaction initiation time and peak velocity (for a schematic depiction of the parameter extraction see Fig 1). We extracted the reaction initiation time from the tracking data by inspecting the reaction speed curves and determined that an approach/avoidance reaction was initiated when surpassing a threshold of 0.10 m/s.

Movement parameters could not be extracted for 0.18% of the trials (21 of 11520) because subjects started the rating procedure before taking a step. Furthermore, we analyzed only trials in which subjects initiated their reaction in the instructed direction, a movement of 10 cm or more in the opposite direction was classified as an incorrect trial, which applied to 3.55% of the remaining trials (408 of 11499). Next, we excluded outliers using the Tukey criterion: We discarded trials 1.5 times the interquartile-range lower than the first or higher than the third quartile across all combinations of avatar sex and reaction direction, separately for each subject and dependent variable (reaction initiation time, peak velocity, and step size). For peak velocity, we thus excluded 2.12% (236 of 11499) and for step size 2.16% (240 of 11499) of the remaining trials. For reaction initiation time, we also excluded RTs under 250ms. This affected 8.56% (949 of 11499) of the remaining trials. Note that this data reduction of about 8% across the dependent variables is a little higher but comparable to other studies [24, 39] in the domain of whole-body approach-avoidance behavior. Considering that we presented double the amount of trials in comparison to previous studies [24, 39], we deem the amount of data reduction unproblematic. The remaining RT data were log_2_-transformed to reduce skewness.

To allow for flexible trial-based modelling of sexual attraction, we analyzed the data using a Bayesian linear mixed model. This approach allows to estimate parameter values of effect sizes and quantify the uncertainty regarding these estimates based on the information in our data and the priors applied. We used brms [40], a wrapper for the STAN-sampler [41], for R to model our data. We applied normally-distributed priors (*M* = 0, *SD* = 1) on all population-level effects, with Cholesky priors on the (residual) correlation (*η* = 2), a *t*-distributed prior (*df* = 3, *M* = 0, *SD* = 1) on the intercept, to allow for thicker tails. The variance parameters were scaled to the respective mean and standard deviation of the target distribution. These priors are only weakly informative and mostly help in the regularization of the posterior distributions. We computed 4 Hamilton-Monte-Carlo chains with 8000 iterations each and 10% warm-up samples. Trace plots of the Markov-chain Monte-Carlo permutations were inspected for divergent transitions. All Rubin-Gelman statistics [42] were well below 1.1, which indicates that the chains converged. We used effect-coding on categorical variables (e.g., .5, -.5).

For statistical inference, following a Bayesian approach, we relied on ($p_{\tilde{b}}$*)* and the high-density posterior intervals (HDI). The posterior median *p*-value was computed by calculating the relative proportion of posterior samples being zero or opposite to the median [for a well-written and accessible introduction see 43]. For an illustration of posterior distributions see Fig 3. Thus, we quantified the proportion of probability that the effect is zero or opposite given the data observed. Note that this is the reverse of the classical approach to inferential statistics, where one measures the probability of the data given the Null-hypothesis with respect to the test statistic. Still, $p_{\tilde{b}}$ should have properties similar to the classical *p*-value [44, 45]. Effects were considered to be meaningful when there was a particular low probability ($p_{\tilde{b}}\leq 2.50\%$) that the effect could be zero or opposite. This threshold was chosen to resemble a conservative two-sided test with an alpha-level of 5%, normally applied in classical statistical inference. In addition to the median of the parameter, we calculated the HDI at 95% of the posterior distribution for all parameters, which indicate the possible range of effects given the data.

To give a standardized estimate of the effects, we calculated δ_*t*_, which can be interpreted quite similar to Cohen’s *d* [46, 47]. Two- or three-way interactions in our model were followed up by posterior predictive tests, which serve a similar purpose as post-hoc comparisons in classical statistical inference. For a reproducible analysis script as well as the data see S1 Code and S1 Data. For regression tables of all population-level effects see S1 Table.

### Results

**Fig 2:**
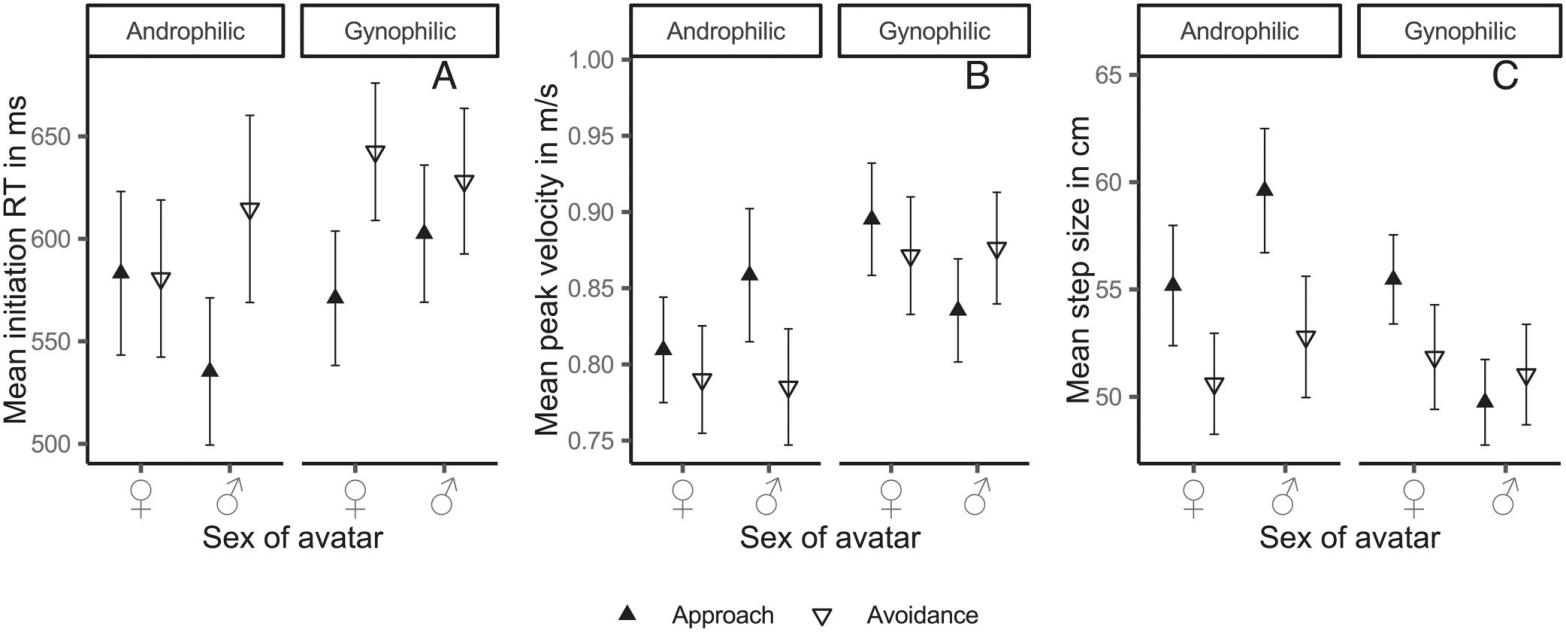
Mean initiation RT (panel A), peak velocity (panel B), and step size (panel C) as a function of sexual orientation, reaction direction, and avatar sex. Error bars denote ±1 standard error of the mean.

Descriptively, androphilic subjects as well as gynophilic subjects approached preferred targets faster and with larger steps, than their non-preferred target, see Fig 2. We modeled the effect of the experimental manipulations on each dependent measure, using a varying intercept for every subject to account for the repeated-measures structure of the data in the mixed model. To allow for individual variation of effects in subjects, we added crossed varying slopes of avatar sex and reaction direction for every subject and a varying intercept with varying slopes for reaction direction for every avatar. The varying intercepts and varying slopes for each subject serve the purpose of normalization and thus controlling for systematic individual differences on the dependent variable (e.g., different length of legs imply different step sizes between subjects, or individual overall variations in reaction time). All population-level effects (i.e., sexual orientation: coded as gynophilic = -.5, androphilic = .5; avatar sex coded as female = .5, male = -.5; and reaction direction: coded as approach = .5, avoidance = -.5) on the outcomes were fully crossed in the model. We first report the results for initiation RT, then for peak velocity and step size.

In total, this model explained ${\tilde{R}}^{2}=59.00\%$ [58.20; 59.81] of the variance of the RT data. There was an effect of reaction direction, $\tilde{b}=-0.10$ [-0.13; -0.07], $p_{\tilde{b}}=0.00\%$, δ_*t*_ = -.17 [-.23; -.11] on initiation RT. On average, approach reactions (*M* = 567 ms, *SD* = 136 ms) were initiated faster than avoidance reactions (*M* = 615 ms, *SD* = 158 ms). As expected, the reaction direction × sexual orientation × avatar sex three-way interaction was well distinguishable from zero, $\tilde{b}=0.22$ [0.04; 0.39], $p_{\tilde{b}}=1.09\%$, δ_*t*_ = 0.37 [0.06;0.66]. Subjects initiated a forward step faster than a backward step when presented with their preferred sex, see Fig 2A.

**Fig 3:**
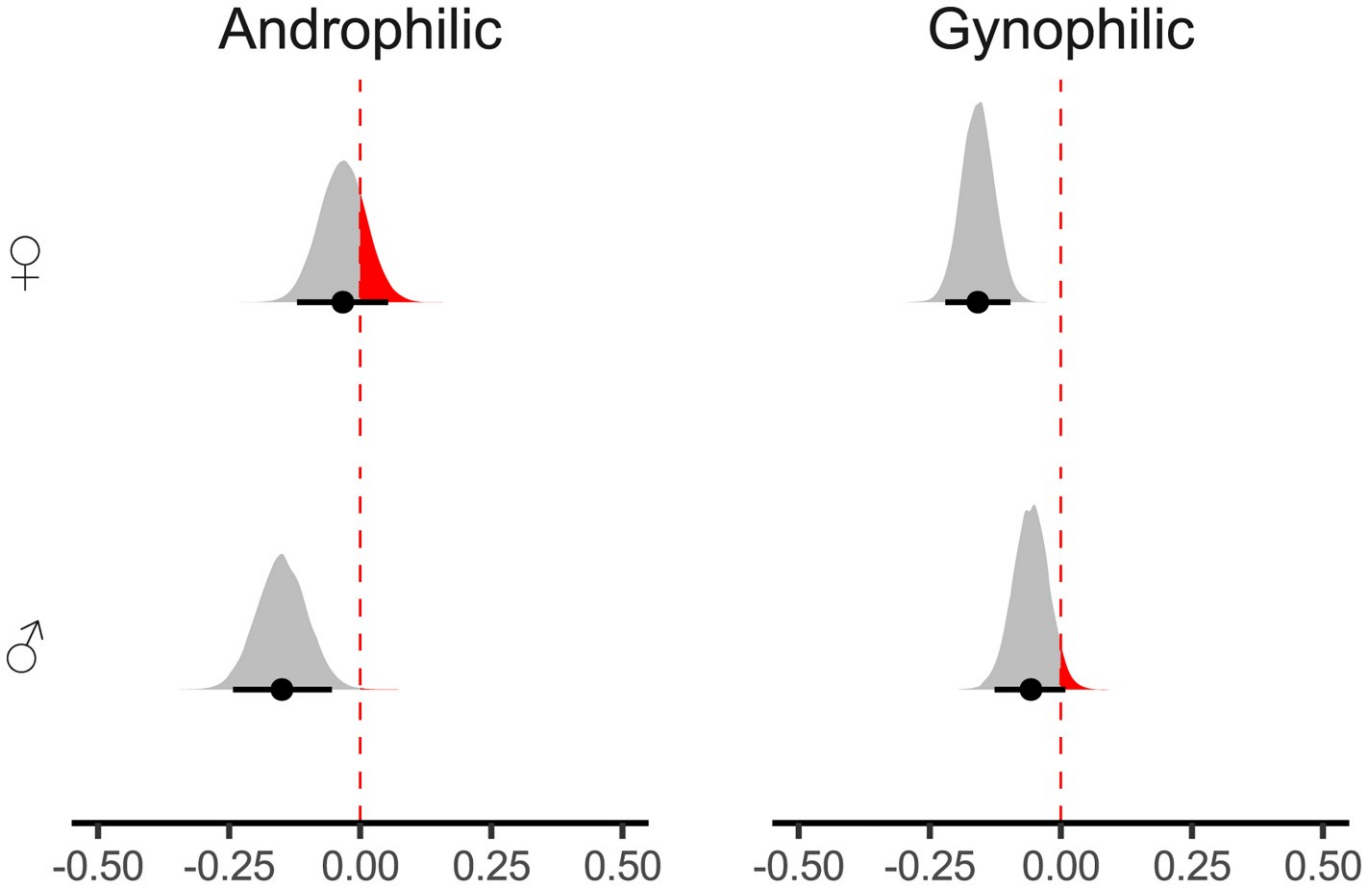
Posterior density plots with 95% high-density intervals, indicated by the error bars, and posterior medians for all approach-biases for reaction initiation time as predicted by the model. The red/black-area indicates the proportion of posterior samples opposite to the median and thus is a visual representation of the posterior median p-value. It quantifies the proportion of probability that the effect is zero or opposite given the data observed. The smaller the red areas are, the more reliable is the estimation of the effect.

All other parameters for initiation RT were approximately zero, all *$p_{\tilde{b}}\geq 5.30$*. We visualized the predicted means using a posterior predictive plot, see Fig 3. This plot depicts an approach-bias, that is a relatively faster approach compared to an avoidance reaction, predicted by our statistical model separated for both sexual orientations and sex of avatar. The descriptive trend towards a facilitation of the average approach reaction for the preferred avatar sex, see Fig 2A, can be also found in our statistical model of initiation RT for both androphilic, $p_{\tilde{b}}=0.16\%$ and gynophilic men, $p_{\tilde{b}}=0.00\%$, see Fig 3.

We fitted the same model to our peak-velocity data, ${\tilde{R}}^{2}=82.28\%$ [82.00; 82.54] and found a slightly different pattern of effects. There was a strong sexual orientation × avatar sex interaction effect, b˜=−0.04ms [-0.06; -0.02], $p_{\tilde{b}}=0.12\%$, δ_*t*_ = -0.10 [-0.16; -0.04]. As shown in Fig 2B, gynophilic subjects produced larger peak velocities for female avatars (*M* = 0.88 ^m^/_s_, *SD* = 0.23 ^m^/_s_) as compared to male avatars (*M* = 0.86 ^m^/_s_, *SD* = 0.21 ^m^/_s_), $p_{\tilde{b}}=0.14\%$. In contrast, the peak velocities of androphilic subjects were not distinguishable from zero, male (*M* = 0.82 ^m^/_s_, *SD* = 0.16 ^m^/_s_) > female (*M* = 0.80 ^m^/_s_, *SD* = 0.15 ^m^/_s_), posterior predictive: $p_{\tilde{b}}=5.92\%$. There was no indication of a three-way interaction of reaction direction × sexual orientation × avatar sex interaction, b˜=−0.05ms [-0.18; 0.09], $p_{\tilde{b}}=23.81\%$, δ_*t*_ = -0.12 [-0.44; .22], concerning the width of the posterior distribution more data may be needed in order to specify the size and direction of the effect. All other parameters were $p_{\tilde{b}}>4.09\%$ with $-0.05>\tilde{b}<0.08$.

Applying this model to the step size data, ${\tilde{R}}^{2}=80.74\%$ [80.44; 81.04], yielded a strong effect of reaction direction,$\tilde{b}=4.29cm$ [1.26; 7.52], $p_{\tilde{b}}=0.33\%$, δ_*t*_ = 0.18 [0.05; 0.32]. Subjects made larger steps forward (*M* = 54.59 cm, *SD* = 13.12 cm) than backward (*M* = 51.69 cm, *SD* = 14.53 cm). This was more pronounced in androphilic subjects,$\tilde{b}=6.55cm$ [0.31; 12.67], $p_{\tilde{b}}=1.79\%$, δ_*t*_ = 0.28 [0.01; 0.54], (Δ androphilic subjects: *M* = -6.28 cm, *SD* = 11.65 cm, posterior predictive: $p_{\tilde{b}}=0.12\%$; Δ gynophilic subjects: *M* = -1.20 cm, *SD* = 12.40 cm, posterior predictive: $p_{\tilde{b}}=28.90\%$). Notably, there was a strong sexual orientation × avatar sex interaction,$\tilde{b}=-6.15cm$ [-8.55; -3.74], $p_{\tilde{b}}=0.00\%$, δ_*t*_ = -0.26 [-0.37; 0.16]. This resembles the effect on peak velocity, i. e., androphilic subjects made larger steps towards male avatars (*M* = 56.82 cm, *SD* = 11.51 cm) as compared to female avatars (*M* = 53.41 cm, *SD* = 11.07 cm, posterior predictive: $p_{\tilde{b}}=0.25\%$), while the reverse pattern emerged for gynophilic subjects who made larger steps toward female avatars (*M* = 54.20 cm, *SD* = 13.72 cm) as compared to male avatars (*M* = 50.83 cm, *SD* = 12.69 cm, posterior predictive: $p_{\tilde{b}}=0.00\%$). The three-way interaction,$\tilde{b}=-4.25cm$ [-10.42; 2.30], $p_{\tilde{b}}=9.88\%$, δ_*t*_ = -0.18 [-0.45; 0.09] as well as the other parameters were centered around zero, $0.17<\tilde{b}>4.17$, $p_{\tilde{b}}>9.67$. Again, more data may be needed in order to estimate the effect with a sufficient level of certainty. Peak velocity and step size were larger when confronted with the preferred avatar sex irrespective of reaction direction.

**Fig 4:**
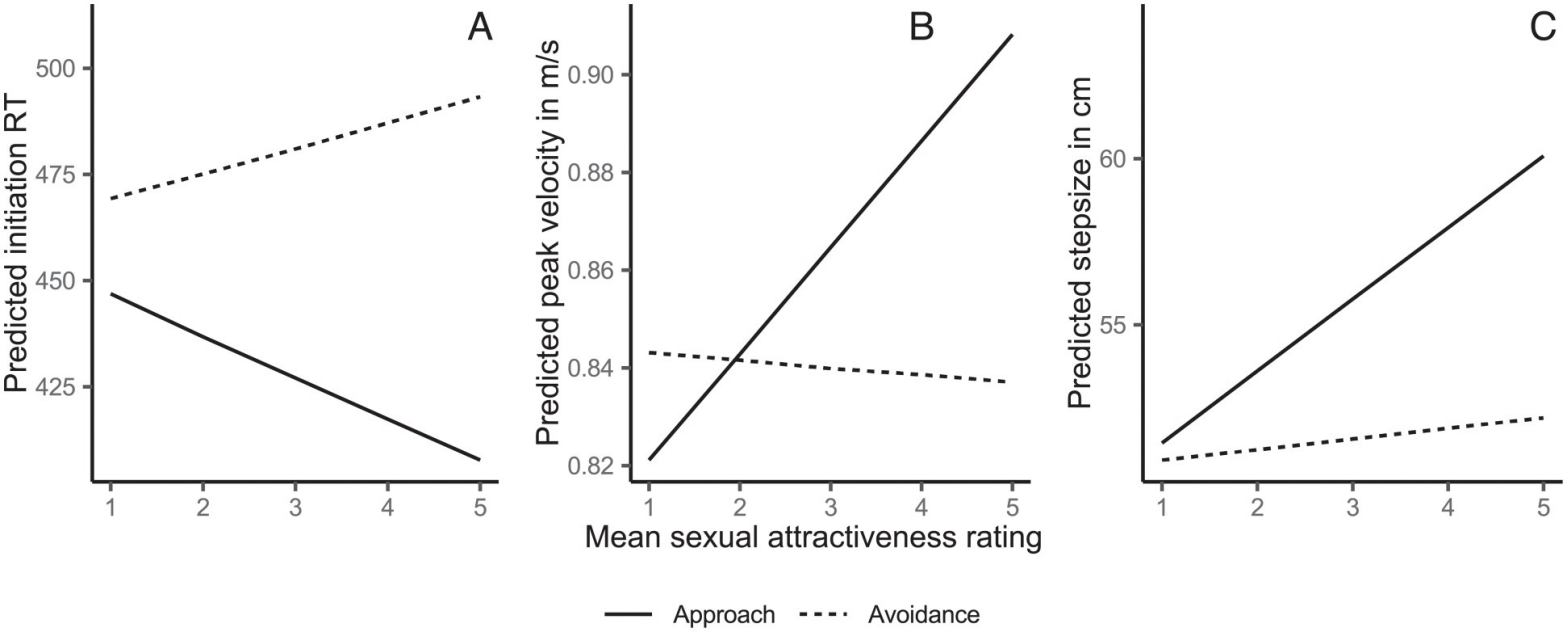
Predicted initiation RT (panel A), peak velocity (panel B), and step size (panel C) as a function of sexual attractiveness (1 = sexually unattractive; 5 = sexually attractive).

In the previous analysis, we had assumed that all gynophilic males were attracted to all female avatars and all androphilic males were attracted to all male avatars in the same way. This implicit model of sexual orientation and sex of avatar, however, does not seem to be appropriate when considering that there is a great variation in ratings of sexual attractiveness (see Fig 4C). This may also partly explain the lack of a three-way interaction of sexual orientation × avatar sex × reaction direction for peak velocity and step size. We chose to construct a more parsimonious and explicit model in which we predicted initiation RT, peak velocity, and step size as a function of reaction direction and sexual attractiveness ratings, which we obtained in each trial (population-level effects). We modeled a varying intercept for every subject and stimulus with varying slopes for reaction direction. This model focused on predicting effects of sexual attractiveness of the avatar on the dependent variables irrespective of sexual orientation and avatar sex.

This more explicit statistical model could explain ${\tilde{R}}^{2}=54.74\%$ [53.84; 55.64] _95% HDI_ for initiation RT. There was a pronounced interaction of sexual attractiveness and reaction direction,$\tilde{b}=-0.04$ [-0.04; -0.03] $p_{\tilde{b}}=0.00\%$, δ_*t*_ = -0.07 [-0.09; -0.05]. The more attractive the avatar, the faster was the approach reaction initiated (see Fig 4A). Note that reactions were initiated faster (i.e., shorter RTs) when avatars were judged to be more attractive (across all reaction directions),$\tilde{b}=-0.01$[-0.01; 0.00], $p_{\tilde{b}}=1.30\%$, δ_*t*_ = -0.01 [-0.02; 0.00].

With regard to the peak velocity data, the model, ${\tilde{R}}^{2}=74.76\%$ [74.33; 75.17], revealed a small direct effect of sexual attractiveness, b˜=−0.01ms [0.01; 0.01], $p_{\tilde{b}}=0.00\%$, δ_*t*_ = 0.03 [0.03; 0.04], which, again, was stronger in approach trials compared to avoidance trials, b˜=−0.02ms [0.02; 0.03], $p_{\tilde{b}}=0.00\%$, δ_*t*_ = 0.03 [0.08; 0.09]. In addition, the model showed generally faster approach reactions as compared to avoidance reactions, b˜=−0.04ms [-0.09; 0.00] $p_{\tilde{b}}=1.49\%$, δ_*t*_ = -0.15 [-0.29; -0.01]. This pattern of results was also found when applying the model to the step size data, ${\tilde{R}}^{2}=75.25\%$ [74.82; 745.65]. Sexually attractive avatars produced larger steps, $\tilde{b}=1.24cm$ [1.12; 1.36], $p_{\tilde{b}}=0.00\%$, δ_*t*_ = 0.09[0.08; 0.11] especially in approach trials, $\tilde{b}=1.84cm$ [1.60; 2.08], $p_{\tilde{b}}=0.00\%$, δ_*t*_ = 0.09 [0.08;0.11].

In sum, we found that higher sexual attractiveness elicited faster (see Fig 4B) and larger (see Fig 4C) steps towards the avatars. Compatible with our hypothesis, the approach reaction was facilitated for attractive avatars across all parameters of the motor reaction. Thus, sexual attraction seems to pull the individual towards the desired stimulus. We examined in the IPD Experiment whether this bias to approach attractive people results in shorter distances within social interactions.

## IPD experiment

The second experiment was designed to study the effect of sexual attraction on IPD and approach speed in virtual encounters. Subjects (andro- and gynophilic males) had to approach an avatar (female vs. male) and had to stop when their preferred IPD for conversation with a stranger was reached. Next, subjects rated the sexual attractiveness of the target.

### Method

We tested the same set of subjects with the same set of stimuli and virtual reality-setup as presented in the AAT Experiment. We varied only one experimental factor within subjects: avatar sex (10 male, 10 female). Each avatar was presented two times, resulting in 40 trials. Trials were presented in random orders. Before the experiment, every subject completed 10 training trials using a set of other avatars. At the beginning of each trial, subjects were presented with a sphere at approximately their own height. After subjects had aimed at the sphere with the controller, the avatar appeared (at a distance of 200 cm) and the subjects engaged in the social interaction (for a recorded video of the procedure see supplementary material; accessible via https://osf.io/w6zxs). They were instructed to walk towards the avatar until a comfortable distance for conversation had been reached for a situation where the subject would have to ask a stranger for directions [for a discussion on this task see 48]. Then, the subject confirmed the position by a button press on the controller and the IPD was logged. Finally, the subject rated the avatar’s sexual attractiveness and then went back to the starting position of 200 cm distance from the avatars.

IPD and peak velocity were the dependent variables extracted from the XYZ data. We collected on average 498 XYZ data points per subject per trial at an average trial duration of 5660 ms. We could not extract peak velocities or IPD for 0.31% of the trials (9 of 2880) because subjects started the rating procedure before moving forward. IPD and peak-velocity measures were corrected for outliers using the Tukey criterion 1.5 times the interquartile-range lower than the first or higher than the third quartile separately for each subject and level of avatar sex. This affected 2.65% (76 of 2871) of the cases for peak velocity and 3.59% (103 of 2871) for IPD. Note that we have also analyzed initiation RT. However, as subjects in the IPD Experiment were not instructed to approach the virtual person as fast as possible, variation in initiation RT could not be modeled properly for this experiment.

### Results

**Fig 5:**
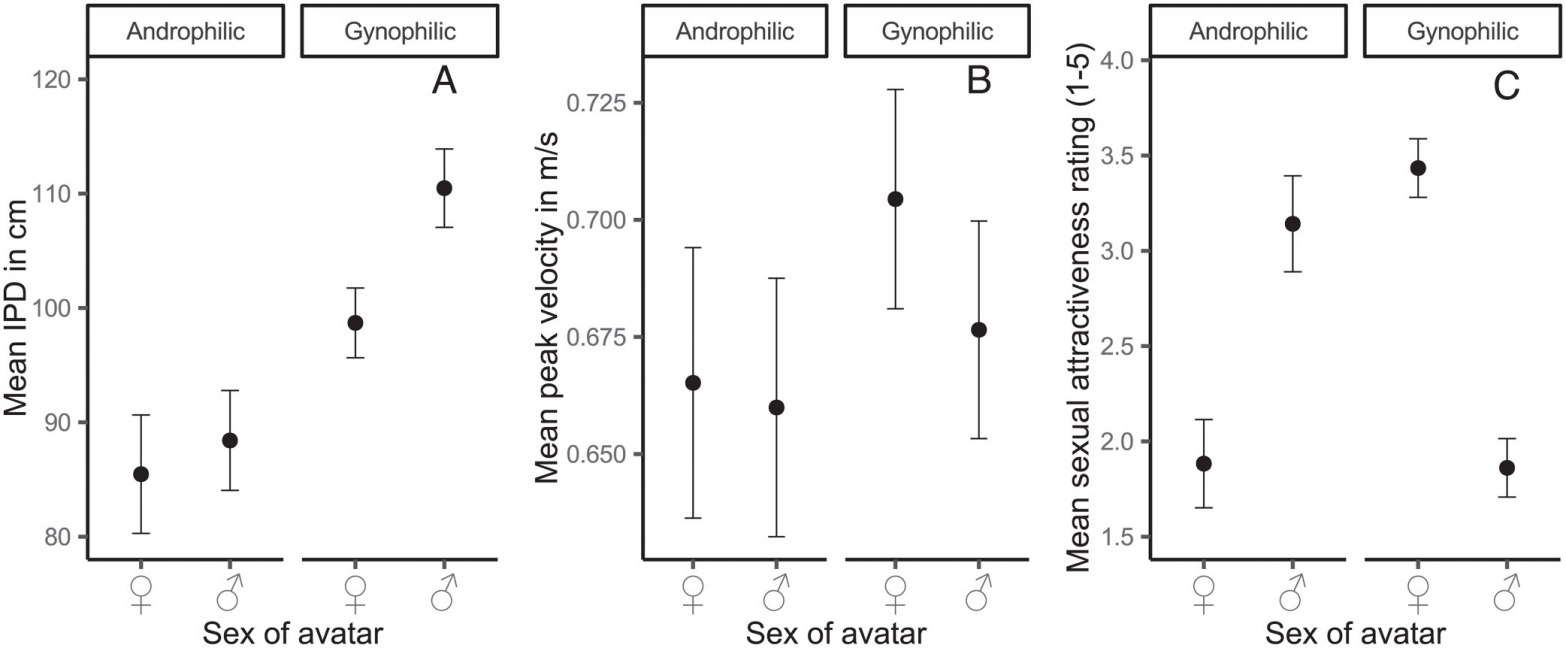
Mean IPD (panel A), peak velocity (panel B), and sexual attractiveness ratings (panel C) as a function of sexual orientation and avatar sex. Error bars denote ±1 standard error of the mean.

In the Bayesian linear mixed model, we estimated a varying intercept for every subject and stimulus with varying slopes for avatar sex to account for the repeated measures structure of the data. All population level effects on the outcomes (sexual orientation and avatar sex) were fully crossed in the model. Thus, all main effects and interaction effects were incorporated into the regression formula. This model explained ${\tilde{R}}^{2}=82.23\%$ [81.66; 82.77] of the variance in the IPD data. Sexual orientation affected IPD. Androphilic subjects preferred shorter distances as compared to gynophilic subjects, which amounted to a reduction of $\tilde{b}=13.15cm$ [-23.01; -3.22] $p_{\tilde{b}}=0.53\%$, δ_*t*_ = -0.50 [-0.89; -0.12]. As expected, there was an effect of avatar sex (male = -0.5, female = 0.5) on IPD, $\tilde{b}=-6.73cm$ [-10.28; -3.13], $p_{\tilde{b}}=0.03\%$, δ_*t*_ = -0.50 [-0.89; -0.12]. Subjects approached female avatars about 7 cm closer than male avatars. Interestingly, this was reduced for androphilic compared to gynophilic males, as indicated by the sexual orientation × avatar sex interaction on IPD,$\tilde{b}=9.84cm$ [3.24; 16.39], $p_{\tilde{b}}=0.14\%$, δ_*t*_ = 0.37[0.12; 0.62]. Thus, as expected, the sex effect on IPD was considerably reduced for androphilic subjects, see Fig 5A. Modelling peak velocity in the same manner, ${\tilde{R}}^{2}=78.82\%$ [78.11; 79.50], yielded only a discernible effect of avatar sex b˜=0.02ms [0.01; 0.03], $p_{\tilde{b}}=0.10\%$, δ_*t*_ = 0.12 [0.04; 0.19]. Female avatars were approached slightly faster than male avatars, see Fig 5B.

Again, to account for individual differences in attractiveness ratings and to see whether sex effects persist when controlling for sexual attractiveness, we modeled IPD and peak velocity as a function of standardized sexual attractiveness and sex of avatar. We estimated a varying intercept for each subject and stimulus to account for the repeated measures structure of the data and added varying slopes sex of avatar in every subject, to allow for individual variation of the sex effect. For IPD, the model explained a comparable proportion of variance, ${\tilde{R}}^{2}=81.38\%$ [80.78; 81.95]. IPD strongly decreased with an increase in the sexual attractiveness ratings,$\tilde{b}=-3.54cm$ [-4.02; -3.04], $p_{\tilde{b}}=0.00\%$, δ_*t*_ = -0.13 [-0.16; -0.11]. Note however, when controlling for sexual attractiveness, the main effect of avatar sex persisted,$\tilde{b}=-7.47cm$ [-11.33; -3.59], $p_{\tilde{b}}=0.01\%$, δ_*t*_ = -0.28 [-0.44; -0.13]. The effect of sexual attractiveness did not vary as a function of gender, $p_{\tilde{b}}=19.17\%$. The same pattern of results was found for peak velocity,${\tilde{R}}^{2}=81.46\%$ [80.85; 82.02]. The model revealed a slight increase in peak velocity when avatars were judged to be sexually attractive, b˜=0.01ms [0.00; 0.01], $p_{\tilde{b}}=0.00\%$, δ_*t*_ = 0.04[0.02; 0.06], and also marginally faster approaches towards female avatars, b˜=0.02ms [0.00; 0.04], $p_{\tilde{b}}=3.30\%$, δ_*t*_ = 0.12[-0.01; 0.24].

Lastly, we investigated whether an approach-bias in the AAT Experiment relates to shorter distances in the IPD Experiment. For the AAT Experiment we calculated *d*-scores only for trials where subjects responded to their preferred avatar sex. We subtracted the mean of approach trials from the mean of avoidance trials and divided this difference by the overall standard deviation of these trials [49], for reaction initiation time, peak velocity, and step distance. Thus, the *d*-scores depict a standardized bias-score to approach rather than to avoid a preferred target.

IPD to the preferred sex was strongly related to an approach-bias in step size. A stronger approach-bias towards avatars of the preferred sex in the AAT Experiment (i. e. larger approach than avoidance steps) was associated with shorter distances towards avatars of the preferred sex in the IPD Experiment. All variables but reaction initiation time were correlated, see Table 1.

**Table 1:** 

	IPD	peak velocity	AAT–RT (*d*)	AAT–peak velocity (*d*)	AAT–step size (*d*)
IPD	-				
peak velocity	-.34*	-			
AAT–RT (*d*)	.16	.06	-		
AAT–peak velocity (*d*)	-.22*	.28*	-.29*	-	
AAT–step size (*d*)	-.35*	.25*	-.26*	-.72*	-

IPD Experiment: Individual mean IPD and peak velocity for the preferred target. AAT Experiment: Individual *d*-scores were computed subtracting approach trials from avoidance trials towards the preferred avatar sex and dividing through the pooled standard deviation for all outcome variables. Correlations with $p_{\tilde{b}}<2.50\%$ are marked with *.

In sum, subjects’ social interaction behavior varied as a function of sexual orientation and avatar sex, probably due to sexual attraction. Sexually attractive avatars produced relatively shorter IPDs. Note, however, that sex effects on IPD persisted when controlling for the effect of sexual attractiveness. At a more explicit level, we could observe that subjects approached attractive targets and female targets relatively faster.

## Discussion

Male subjects seem to be attracted towards virtual persons who match their sexual orientation. In the AAT Experiment, subjects initiated approach movements towards avatars of their preferred sex faster than they initiated avoidance movements. No such difference was found for the non-preferred sex. Here, instructed approach and avoidance were initiated in the same manner. Subjects also made larger and faster steps in response to their preferred sex avatar. This pattern of effects can be explained when considering sexual attractiveness. The facilitation of approach steps was related to the individual degree of rated sexual attractiveness.

The IPD Experiment showed that gynophilic males preferred shorter IPDs towards female avatars as compared to male avatars, which is consistent with previous studies [e.g. 20]. However, the effect of avatar sex on IPD was considerably diminished in androphilic males. Furthermore, androphilic subjects preferred shorter overall IPDs compared to gynophilic subjects. Similarly to the sex effect on IPD, the peak velocity of the approach reaction was faster towards female avatars as compared to male avatars in gynophilic subjects. These differences were not present in androphilic subjects.

What fuels this behavior? As proposed by the Dual Control Model and related theories in sex research, sexual arousal caused by sexual attraction promotes approach tendencies (AAT Experiment), which results in shorter and more intimate conversation distances (IPD Experiment). It is remarkable that these effects do carry over to virtual avatars.

In the second Experiment, IPD varied as a function of sexual orientation when interacting with male and female virtual persons. Gynophilic subjects produced the well-known sex effect on IPD, i. e., shorter distances towards females as compared to males; interestingly, this effect was absent in androphilic subjects. The diminished sex effect on IPD in androphilic subjects could be interpreted in light of equilibrium theory [14, 21]: Sexual attraction to men promotes approach tendencies (see AAT Experiment) towards male avatars, but no avoidance towards female avatars, which merely reduces the preferred IPD to male avatars, thus resulting in equal IPD between male and female avatars. The same reasoning applies to the differential sex effect on peak velocity as effects are largely correlated within the IPD Experiment.

When correlating the step size-bias from the AAT Experiment and preferred distance from the IPD Experiment, we could find a medium-sized correlation between the two aggregates, which again strengthens the hypothesis that IPD is regulated by approach and avoidance forces [14, 21]. A recent study by Ruggiero, Rapuano [50] lends credibility to this interpretation, i.e. approach motivation promoting smaller IPD. They found that inducing warmth by holding a warm beverage, which was supposed to increase approach motivation, produced smaller IPD than did the induction of coldness.

Note that when controlling for sexual attraction, the sex effect on IPD persisted. Referring to Uzzell and Horne [17], our findings suggest that sexual orientation and thus sexual attraction may partly overshadow the sex effect on IPD. Previous studies investigating sex effects [17, 19, 20] have presented a small set of targets, have not measured explicit sexual attractiveness and sexual orientation, or did not use an immersive social interaction scenario, which limited the ability to detect sex effects on IPD and disentangle the effect from sexual attractiveness. Furthermore, although Uzzell and Horne [17] did find an effect of sexual orientation on IPD, they could not reveal any differences between androphilic and gynophilic men, probably due to low statistical power and experimental control. Therefore, our study can be considered the first study to show an interaction of sexual orientation and sex of the approached person on IPD in men. Our study can also reveal why sex effects on IPD tend to be heterogeneous. Sexual attractiveness can serve as an important determinant of IPD and may therefore override sex effects. As this depends on the degree of attractiveness, it could explain the heterogeneous results across studies, e.g. when a confederate is particularly attractive in a social interaction task measuring IPD and/or when androphilic subjects make up a proportion of the sample.

Note also that we did not expect on overall difference in IPD between gynophilic and androphilic male subjects. This is in line with Uzzell and Horne [17] who report an overall similar pattern of IPD preferences in their observational study (gynophilic > androphilic males; by about 6 cm). Thus, this difference in preferred IPD between androphilic and gynophilic male subjects deserves to be further investigated with a larger sample powered to study overall between-subjects variability.

This is the first study to investigate a whole-body approach and avoidance movements within a virtual environment. The AAT reliably detected effects on different sets of measurements and converged with the results in the IPD Experiment. Furthermore, we found effects on a range of dependent variables, which adds credibility to the smaller effects found in previous AAT studies [7, 10]. These results indicate that the AAT in combination with the IPD-paradigm may be more ecologically valid than alternative approaches used in previous studies (e. g., using 2D-stimuli and arm-movements). Thus, we believe that these tasks may also be useful in other fields of research with a focus on approach/avoidance-behavior, such as social interaction behavior in psychopathology or forensic research.

In the domain of sex research, our data provide further evidence for the assumption that sexually arousing stimuli do not solely activate sexually specific motor responses but also general locomotor approach behavior [51]. We found that sexually relevant stimuli affect IPD in virtual social encounters. Thus, we assume that the propensity of reacting on stimuli perceived as sexually relevant may influence our every-day social interaction behavior. In this regard, our study points to the potential of using VEs within sex research. Contrary to previous research, we could observe social interaction behavior with respect to sexual attraction in an ecologically more valid and highly controlled fashion. Subjects were instructed to approach a virtual person as if they wanted to ask for directions, which is potentially more ecologically valid than current explicit and implicit measures of sexual interest [for an overview see 52]. For viewing time, an implicit measure of sexual interest, it has already been shown that presenting virtual characters in highly immersive environments can enhance the discriminative validity of viewing time [33].

These findings are also potentially useful for forensic psychology. Sexual motivation is a key component in recent models of sexual offense behavior [53–56]. Our results may be applied to a sample of people who have sexually offended, in order to measure the strength of their approach reaction and to distinguish approach- vs. avoidance-oriented individuals who have committed sexual offenses, to allocate treatment resources more appropriately and efficiently. Some limitations must be considered with reference to our sample and to our method. First, we have only studied male western subjects. Future studies should replicate our findings in a female sample and include non-western subjects. This is particularly important considering the variation between nationalities [29, 30] which could potentially slightly enhance or diminish the sex effect on IPD. Second, we have not controlled for sexual identity [17]. Masculinity or femininity could also influence the sex effect on IPD.

Third, we have confronted subjects with a larger number of trials in a relatively small amount of time (230 trials in 90 minutes). We have also administered the experiments in a fixed order. The IPD Experiment was always followed by the AAT Experiment to minimize potential effects of familiarity in the IPD Experiment. Both factors could have contributed to fatigue, habituation to the stimuli as well as exhaustion due to our request for rapid stepping movement, especially in the AAT Experiment. This could reduce the magnitude of the effects and should be considered in future studies as a possible enhancement. We hypothesize that a randomized order will not change the direction of the reported effects, which may be evaluated in future studies with larger samples. Still, before application of the AAT or the IPD-paradigm in applied forensic contexts, the length of data collection as well as the task demands should be carefully evaluated and reduced. Fourth, administering the AAT in a virtual environment is a new measure that deserves further investigation in terms of reliability and validity.

In conclusion, the recording of IPD-regulation and the approach-avoidance scenario, both implemented in a virtual environment, provide a powerful and rather implicit paradigm to study the effects of sexual attractiveness on behavioral propensities.

## Supporting information

S1 Data Data for the AAT Experiment and the IPD Experiment as well as relevant demographics. This file provides an R image of all relevant Dataframes and functions.
(RDS)

S1 Code Analysis script. This R-script provides a commented syntax for data preparation, model-fitting, analysis of the posterior and data visualization.
(R) Click here for additional data file.

S1 Table Regression tables for population-level effects of all models. (DOCX)
