# Correction: Sexual attraction modulates interpersonal distance and approach-avoidance movements towards virtual agents in males

**DOI:** 10.1371/journal.pone.0239935

**Published:** 2020-09-24

**Authors:** The PLOS ONE Staff

## Notice of Republication

Incorrect versions of Fig 3 and the Supporting Information files were published in error. This article was republished on May 18, 2020, to correct for this error. The publisher apologizes for the errors. Please download this article again to view the correct version.
