# Supplementary material for: Sexual attraction modulates interpersonal distance and approach-avoidance movements towards virtual agents in males
Source: PLoS One. 2020 Apr 21;15(4):e0231539. doi: 10.1371/journal.pone.0231539 (PMC7173797; doi:10.1371/journal.pone.0231539)
Supplement: S1 Table — (DOCX) [file pone.0231539.s003.docx]

**Regression tables for population-level effects of all models**

**AAT Experiment**

**S1 Table 1. Population-level effects for RT concerning the model with Sexual orientation, Avatar sex and reaction direction for the AAT Experiment**

| Parameter | Median Estimate | Std.Error | HDI(95%) | *p_b̃_* |
| --- | --- | --- | --- | --- |
| Intercept | -0.86 | 0.04 | [-0.94, -0.77] | 0.00% |
| Sexual orientation | -0.12 | 0.08 | [-0.30, 0.04] | 8.17% |
| Avatar sex | -0.01 | 0.01 | [-0.03, 0.02] | 30.90% |
| Reaction direction | -0.10 | 0.02 | [-0.13, -0.07] | 0.00% |
| Sexual orientation:Avatar sex | 0.03 | 0.02 | [-0.01, 0.07] | 5.30% |
| Sexual orientation: Reaction direction | 0.02 | 0.03 | [-0.05, 0.08] | 30.71% |
| Avatar sex: Reaction direction | 0.01 | 0.05 | [-0.08, 0.10] | 43.72% |
| Sexual orientation:Avatar sex: Reaction direction | 0.22 | 0.09 | [ 0.04, 0.39] | 1.09% |

**S1 Table 2. Population-level effects for peak velocity concerning the model with Sexual orientation, Avatar sex and reaction direction for the AAT Experiment**

| Parameter | Median Estimate | Std.Error | HDI(95%) | *p_b̃_* |
| --- | --- | --- | --- | --- |
| Intercept | 0.85 | 0.03 | [ 0.79, 0.90] | 0.00% |
| Sexual orientation | -0.02 | 0.05 | [-0.13, 0.08] | 32.84% |
| Avatar sex | 0.00 | 0.01 | [-0.01, 0.02] | 29.53% |
| Reaction direction | 0.02 | 0.02 | [-0.02, 0.06] | 15.03% |
| Sexual orientation:Avatar sex | -0.04 | 0.01 | [-0.06, -0.02] | 0.12% |
| Sexual orientation: Reaction direction | 0.08 | 0.04 | [-0.01, 0.16] | 4.09% |
| Avatar sex: Reaction direction | 0.03 | 0.03 | [-0.04, 0.10] | 18.02% |
| Sexual orientation:Avatar sex: Reaction direction | -0.05 | 0.07 | [-0.18, 0.09] | 23.81% |

**S1 Table 3. Population-level effects for step size concerning the model with Sexual orientation, Avatar sex and reaction direction for the AAT Experiment**

| Parameter | Median Estimate | Std.Error | HDI(95%) | *p_b̃_* |
| --- | --- | --- | --- | --- |
| Intercept | 53.86 | 1.62 | [ 50.51, 56.97] | 0.00% |
| Sexual orientation | 4.17 | 3.33 | [ -2.04, 10.84] | 9.67% |
| Avatar sex | 0.17 | 0.65 | [ -1.10, 1.48] | 39.82% |
| Reaction direction | 4.29 | 1.57 | [ 1.26, 7.52] | 0.33% |
| Sexual orientation:Avatar sex | -6.15 | 1.22 | [ -8.55, -3.74] | 0.00% |
| Sexual orientation: Reaction direction | 6.55 | 3.10 | [ 0.31, 12.67] | 1.79% |
| Avatar sex: Reaction direction | 1.88 | 1.61 | [ -1.45, 4.99] | 12.23% |
| Sexual orientation:Avatar sex: Reaction direction | -4.25 | 3.22 | [-10.42, 2.30] | 9.88% |

**S1 Table 4. Population-level effects for RT concerning the model with Sexual attractiveness and reaction direction for the AAT Experiment**

| Parameter | Median Estimate | Std.Error | HDI(95%) | *p_b̃_* |
| --- | --- | --- | --- | --- |
| Intercept | -0.82 | 0.04 | [-0.91, -0.74] | 0.00% |
| Sexual attractiveness | -0.01 | 0.00 | [-0.01, -0.00] | 1.30% |
| Reaction direction | -0.01 | 0.02 | [-0.05, 0.03] | 24.73% |
| Sexual attractiveness: Reaction direction | -0.04 | 0.00 | [-0.04, -0.03] | 0.00% |

**S1 Table 5. Population-level effects for peak velocity concerning the model with Sexual attractiveness and reaction direction for the AAT Experiment**

| Parameter | Median Estimate | Std.Error | HDI(95%) | *p_b̃_* |
| --- | --- | --- | --- | --- |
| Intercept | 0.82 | 0.02 | [ 0.77, 0.87] | 0.00% |
| Sexual attractiveness | 0.01 | 0.00 | [ 0.01, 0.01] | 0.00% |
| Reaction direction | -0.04 | 0.02 | [-0.09, -0.00] | 1.49% |
| Sexual attractiveness: Reaction direction | 0.02 | 0.00 | [ 0.02, 0.03] | 0.00% |

**S1 Table 6. Population-level effects for step size concerning the model with Sexual attractiveness and reaction direction for the AAT Experiment**

| Parameter | Median Estimate | Std.Error | HDI(95%) | *p_b̃_* |
| --- | --- | --- | --- | --- |
| Intercept | 49.92 | 1.49 | [46.91, 52.90] | 0.00% |
| Sexual attractiveness | 1.24 | 0.06 | [ 1.12, 1.36] | 0.00% |
| Reaction direction | -1.30 | 1.55 | [-4.27, 1.70] | 19.95% |
| Sexual attractiveness: Reaction direction | 1.84 | 0.12 | [ 1.60, 2.08] | 0.00% |

**IPD Experiment**

**S1 Table 7. Population-level effects for stepsize concerning the model with Sexual orientation and Avatar sex for the IPD Experiment**

| Parameter | Median Estimate | Std.Error | HDI(95%) | *p_b̃_* |
| --- | --- | --- | --- | --- |
| Intercept | 95.68 | 2.52 | [ 90.65, 100.99] | 0.00% |
| Sexual orientation | -13.15 | 5.01 | [-23.01, -3.22] | 0.53% |
| Avatar sex | -6.73 | 1.80 | [-10.28, -3.13] | 0.03% |
| Sexual orientation:Avatar sex | 9.84 | 3.32 | [ 3.24, 16.39] | 0.14% |

**S1 Table 8. Population-level effects for peak velocity concerning the model with Sexual orientation and Avatar sex for the IPD Experiment**

| Parameter | Median Estimate | Std.Error | HDI(95%) | *p_b̃_* |
| --- | --- | --- | --- | --- |
| Intercept | 0.68 | 0.02 | [ 0.65, 0.72] | 0.00% |
| Sexual orientation | 0.01 | 0.04 | [-0.06, 0.08] | 40.78% |
| Avatar sex | 0.02 | 0.01 | [ 0.01, 0.03] | 0.10% |
| Sexual orientation:Avatar sex | -0.02 | 0.01 | [-0.04, 0.01] | 9.35% |

**S1 Table 9. Population-level effects for peak velocity concerning the model with Sexual attractiveness and Avatar sex for the IPD Experiment**

| Parameter | Median Estimate | Std.Error | HDI(95%) | *p_b̃_* |
| --- | --- | --- | --- | --- |
| Intercept | 106.97 | 2.58 | [101.83, 112.20] | 0.00% |
| Sexual attractiveness | -3.54 | 0.25 | [ -4.02, -3.04] | 0.00% |
| Avatar sex | -7.47 | 1.94 | [-11.33, -3.59] | 0.01% |
| Sexual attractiveness:Avatar sex | 0.46 | 0.52 | [ -0.56, 1.43] | 19.17% |

**S1 Table 10. Population-level effects for peak velocity concerning the model with Sexual attractiveness and Avatar sex for the IPD Experiment**

| Parameter | Median Estimate | Std.Error | HDI(95%) | *p_b̃_* |
| --- | --- | --- | --- | --- |
| Intercept | 0.66 | 0.02 | [ 0.63, 0.70] | 0.00% |
| Sexual attractiveness | 0.01 | 0.00 | [ 0.00, 0.01] | 0.00% |
| Avatar sex | 0.02 | 0.01 | [-0.00, 0.04] | 3.30% |
| Sexual attractiveness:Avatar sex | 0.00 | 0.00 | [-0.01, 0.01] | 45.50% |
